# Fluorescence in situ hybridization in 1 mL of selective urine for the detection of upper tract urothelial carcinoma: a feasibility study

**DOI:** 10.1007/s12032-018-1237-x

**Published:** 2018-11-29

**Authors:** J. E. Freund, E. I. M. L. Liem, C. D. Savci-Heijink, T. M. de Reijke

**Affiliations:** 1 Department of Urology, Amsterdam University Medical Centers, University of Amsterdam, Meibergdreef 9 G4-223, 1105AZ Amsterdam, The Netherlands; 2 Department of Pathology, Amsterdam University Medical Centers, University of Amsterdam, Meibergdreef 9, 1105AZ Amsterdam, The Netherlands

**Keywords:** Fluorescence in situ hybridization, Urothelial carcinoma, Upper tract urothelial carcinoma, Urine cytology, Ureteroscopy, Cystoscopy

## Abstract

Kidney-sparing surgery of upper tract urothelial carcinoma (UTUC) requires a stringent follow-up with frequent ureteroscopies. Triage testing could reduce the number of follow-up ureteroscopies and hence minimize the invasiveness of follow-up. The use of urine-based markers for triage seems appealing but should be feasible with selective urine from outpatient cystoscopy to maximize the reduction of invasiveness. In this study, the feasibility of UroVysion^®^ fluorescence in situ hybridization (FISH) for the detection of UTUC in 1 mL of selective urine is investigated. Ten consecutive patients with biopsy-proven UTUC and five patients with negative diagnostic ureteroscopy findings were included in this case-control study. During ureteroscopy, 1 mL of selective urine was collected passively with a ureteral splint for Urovysion® FISH. The FISH rater was blinded to any clinical information. The results of FISH were compared to the findings of concomitantly collected selective urine cytology and the patients’ UTUC status. FISH was feasible in all samples with a sensitivity of 90% and a specificity of 80% for UTUC. In comparison, selective cytology resulted in a diagnostic yield of 87% with a sensitivity of 80% and a specificity of 67%. In conclusion, UTUC detection is feasible with FISH in 1 mL of passively collected selective urine. Thus from a technical point of view, FISH could be used as an outpatient triage test to decide if follow-up ureteroscopy is necessary after kidney-sparing surgery of UTUC. Evaluation of the diagnostic accuracy of FISH for the suggested pathway deserves further attention.

## Introduction

Upper urinary tract urothelial carcinoma (UTUC) arises from the urothelial lining of the ureter and pyelocalyceal system. The peak incidence of the disease is at 70–90 years of age. Ureterorenoscopy (URS) under general anesthesia is the corner stone for UTUC diagnosis. URS enables the visualization of the upper urinary tract (UT) urothelium and the acquisition of tissue biopsies. Histopathologic assessment of suspicious lesions is essential for primary diagnostics and risk-stratification for adequate treatment selection [1].

High-risk UTUC is generally treated by radical nephroureterectomy (RNU). In low-risk disease (per definition low-grade UTUC), kidney-sparing surgery (KSS) is the primary choice of treatment [1]. KSS may be offered in the form of ureteroscopic laser fulguration or segmental ureterectomy for ureteral tumors. These treatment options yield a similar cancer specific survival as RNU in low-risk cases [1]. However, high local recurrence rates after KSS are a point of concern that asks for a stringent follow-up [1]. Optimal follow-up includes frequent URS under general anesthesia, which is demanding for the elderly UTUC population. Triaging before follow-up URS could lead to a reduction in the number of invasive diagnostic interventions and hence improvements in the quality of life and reduction of health care costs after KSS.

Urine-based diagnostics are an appealing option for triage testing in the follow-up after KSS. However, standard cytological assessment of selectively collected urine from the upper urinary tract is not suitable as a triage test for the suggested pathway. This is because selective urine cytology lacks sensitivity for low-grade UTUC and KSS is especially offered to low-grade disease [2–4]. Yet, specific urine-based markers could be more suitable for triage testing in the follow-up after KSS.

UroVysion® FISH is an approved urine-based cytogenetic test for the detection of urothelial carcinoma of the bladder (UCB) [5]. Several studies have already investigated the role of FISH in diagnosing UTUC and reported promising results [6–8]. As such, UroVysion^®^ FISH may qualify as a triage test for the follow-up after KSS. However, as the manufacturer advices a minimum urine volume of 32 mL for FISH analysis, the feasibility of FISH in small volumes of selective urine that can be collected passively during outpatient cystoscopy from the upper urinary tract has not been explored yet.

In the present study, the feasibility of UroVysion^®^ FISH for the detection of UTUC in 1 mL of passively collected selective upper tract urine is investigated. To our knowledge, this is the first study that assessed the use of UroVysion^®^ FISH in passively collected upper tract urine in a manner that could be applied during outpatient cystoscopy without general anesthesia. Additionally, the results of FISH are compared with the findings of standard cytology from concomitantly collected upper urinary tract urine.

## Methods

### Patients and sampling

From September 2017 until March 2018, ten consecutive patients with histopathological confirmed UTUC and five consecutive patients with negative findings during URS were included prospectively in this case–control study.

The UTUC group consisted of adult patients who underwent flexible digital URS (Olympus V2 or Karl Storz Flex XC) with ureteroscopic biopsies of UTUC and selective urine cytology sampling for standard clinical care. The presence of UTUC was confirmed by ureteroscopic biopsies. Biopsies were taken with Piranha^®^ forceps or French Zero-tip baskets (both Boston Scientific, Massachusetts, USA). Histopathologic assessment was performed according to the local clinical protocol by an uropathologist (CDS), who was blinded for FISH results.

The control-group consisted of adult patients in whom the presence of UTUC was ruled out by visual assessment of the complete upper urinary tract by flexible digital URS (Olympus V2 or Karl Storz Flex XC). Selective urine cytology sampling was performed for standard clinical care.

Patients were excluded in case of bladder cancer within 3 months prior to or at the time of URS with selective urine sampling. Additionally, patients were excluded for the control-group if UTUC was present in either UT within 3 years prior to the selective urine sampling.

The institutional review board granted a waiver for this case–control feasibility study as no additional activities in human subjects were involved.

### Procedure

Urine sampling was performed under general anesthesia in the operation theater at the start of the diagnostic URS. According to the standard clinical protocol, patients received 20 mg furosemide, intravenously approximately 20 min prior to urine sampling to stimulate urine production. Selective urine sampling was performed passively via a six French ureteral splint, which was placed under fluoroscopy through a rigid cystoscope (Olympus) or semirigid ureteroscope (Karl Storz) in the distal/midureter. 1 mL of the sampled UT urine was used for FISH analysis and the remaining volume of at least 3 mL was used for standard cytological assessment. Contrast-based retrograde fluoroscopy and introduction of the ureteroscope were performed after the described urine sampling had been completed.

The FISH sample was immediately mixed with 0.5 mL Carbowax (polyethylene glycol) for fixation. Cytospins were made within 24 hours, which resulted in two slide preparations per sample. The slide preparations were fixed with Carnoy’s solution (3:1 methanol:glacial acetic acid) and stored at − 20 °C until FISH was performed.

Selective urine cytology was processed and assessed according to the standard clinical protocol by a cyto-technologist and uropathologist, both blinded for FISH results.

### FISH protocol and interpretation

All slide preparations were analyzed using the UroVysion® FISH bladder cancer assay (Abbott Molecular, Illinois, USA). This FISH assay enables the visualization of molecular alterations (aneuploidy of chromosome 3, 7, and 17; loss of locus 9p21) commonly seen in UCB. The pre-mixed and pre-denatured UroVysion® probe mixture consists of four fluorescent labeled nucleic acid probes [Chromosome Enumeration Probe (CEP) 3, CEP 7, CEP 17, and locus specific identifier (LSI) 9p21]. With excitation of the hybridized probes, the number of the specific chromosome copies is visualized for enumeration.

The slides were pre-treated with the UroVysion^®^ Vysis pre-treatment kit. FISH was performed according to the manufacturer’s protocol with overnight hybridization of the probe mixture with the ThermoBrite System (Abbott) (2 min at 73 °C, 12–16 h at 37 °C). For post-hybridization, the slide preparations were washed in 2X SSC/0.1% NP-40 at room temperature until the coverslips were floated off, 0.4X SSC/0.3% NP-40 at 72 °C for 2 min, and lastly again in 2X SSC/0.1% NP-40 at room temperature for 1 min. The nuclei were counterstained with DAPI (40,6-diamidino-2-phenylindole).

A single, trained observer (JEF), who was blinded for clinical data, performed enumeration of FISH signals. For enumeration, a fluorescence microscope (Leica DM 5500B) was used with the prescribed filters: A4 blue for DAPI, TX2 red for CEP 3, L5 green for CEP 7, SAQ aqua for CEP 17, and SGO gold for LSI 9p21. Specimens were considered to be positive for FISH if among 25 morphologically abnormal cells (large nuclear size, irregular nuclear shape, patchy DAPI staining) ≥ 4 cells had a gain of 2 or more chromosomes (3, 7, or 17), or ≥ 12 cells had a loss of both copies of LSI 9p21 [9].

## Results

The patient characteristics of the UTUC group and the control-group are presented in Table 1. Table 2 lists the distribution of FISH and cytology findings of the UTUC group and the control-group. Sufficient cells were present in all 15 FISH preparations for FISH enumeration, resulting in a diagnostic yield of 100%. The diagnostic yield for selective urine cytology was 87% as two of the 15 UT cytology samples were interpreted as inconclusive.

**Table 1:** Patient characteristics per group

	UTUC group (*n* = 10)	Control-group (*n* = 5)
Gender: women/men	3/7	2/3
Age in years, median (range)	70 (46–90)	61 (39–71)
Prior UTUC history, n	6	3
Highest grade of former UTUC (WHO 2004) (*n*)		
Low-grade	1	2
High-grade	5	1
Prior UCB (*n*)	5	1
Highest grade of former UCB (WHO 2004) (*n*)		
Low-grade	3	1
High-grade	2	0
Time since last UCB in months, median (range)	72 (4–86)	166 (0)

*UCB* urothelial carcinoma of the bladder, *UTUC* upper tract urothelial carcinoma

**Table 2:** Findings per case

Case	Tumor location	Histologic grade from biopsy (WHO 2004)	Selective urine cytology finding	FISH interpretation
1	Distal ureter	HG	No malignancy	+
2	Upper pole and interpolar	HG	HG	+
3	Upper pole	LG	LG	+
4	Renal pelvis	LG	LG	+
5	Renal pelvis	LG	HG	+
6	Distal ureter and renal pelvis	HG	HG	+
7	Distal ureter	HG	HG	+
8	Upper pole	HG	HG	+
9	Renal pelvis	LG	No malignancy	−
10	Distal ureter	HG	LG	+
11	No UTUC visualized	Not applicable	Inconclusive	−
12	No UTUC visualized	Not applicable	Inconclusive	−
13	No UTUC visualized	Not applicable	LG	−
14	No UTUC visualized	Not applicable	No malignancy	+
15	No UTUC visualized	Not applicable	No malignancy	−

*HG* high-grade, *LG* low-grade, + positive for UTUC, − negative for UTUC

As shown in Table 2, nine of the ten FISH assays were positive (sensitivity 90%) in the UTUC group, while eight of ten selective urine cytology findings were positive for low-, or high-grade UTUC (sensitivity 80%). In the control-group, four FISH assays were negative, yielding a specificity of 80%. For selective urine cytology, two of three conclusive findings were negative for UTUC, resulting in a specificity of 67%.

## Discussion

UTUC detection is feasible with UroVysion^®^ FISH in one mL of selective urine. This enables FISH to detect UTUC in small volumes of UT urine that could be collected passively via a ureteral splint during outpatient cystoscopy. From a technical point of view, FISH may, therefore, qualify as a triage test in the outpatient setting to reduce the number of URS in the follow-up after KSS. Limiting the number of invasive URS in the follow-up after KSS is desirable for quality of life improvements in the primarily elderly population of UTUC. Moreover, reducing surgical follow-up under general anesthesia with hospitalization might also reduce procedure-associated complications and health care costs.

Yet, the implementation of FISH as a triage test is dependent on its diagnostic accuracy. In this study, the diagnostic accuracy seems promising but is preliminary. Comparison with the current literature seems somewhat arbitrary when taking into account the different cut-off values and various types of urine sampling methods investigated (voided, UT brushing, UT washing, and passively collected UT urine). Nevertheless, the sensitivity and specificity of FISH for UTUC detection in this feasibility study are in line with the reported range in the literature (35–88% and 78–96%, respectively) [6, 10–13]. We believe that further studies to investigate the diagnostic accuracy of FISH as a triage test for ureteroscopy in the follow-up after KSS of UTUC are warranted.

In the present study, the diagnostic accuracy of FISH seems superior to the diagnostic accuracy of selective UT urine cytology for UTUC detection. Also in the literature, urine cytology for UCB and UTUC is known to have a lower sensitivity at a comparable or higher specificity than FISH [4, 13–16]. But, especially for low-grade urothelial carcinoma, the diagnostic yield of urine cytology is limited [4]. This hampers the suitability of standard cytological assessment as a triage test for the follow-up after KSS, which is generally performed in low-grade UTUC only. Furthermore, challenges in distinction of reactive and neoplastic causes of atypia lead to inaccurate reporting of urine cytology [17]. Inter-observer variability may also influence the diagnostic accuracy of both urine-based tests. Despite the fact that the interpretation of FISH is dependent to subjectivity, FISH results may be more quantifiable than cytology [18].

A limitation of the current study is the small sample size and the case-control study design. This might lead to an inaccurate estimation of the diagnostic accuracies. Due to the highly selected patient cohort, the influence of concomitant UCB on FISH results and cytology findings remains to be investigated. In case of concomitant UCB, ureteral reflux of UCB cells may lead to higher false positive rates [8]. Moreover, the role of ureter splint location for selective urine collection with regard to ureteral reflux and tumor location has not been identified yet.

Next, FISH interpretation was only performed according to the manufacturer’s instructions. Interpretation with cut-off values for enumeration other than specified by the manufacturer might yield different diagnostic accuracies for UTUC detection [19]. Additionally, the lack of multiple raters does not facilitate the assessment of inter-observer variability of FISH interpretations or diagnostic accuracy range calculations.

Likewise, new and potentially superior interpretation methods for reporting urine cytology, such as The Paris System (TPS), were not included in this study. Consequently, the diagnostic accuracy of selective urine cytology might be underestimated when considering the promising results with TPS [20].

To identify the possible role of FISH for the follow-up of KSS, the diagnostic accuracy of FISH in selective urine from outpatient cystoscopy should be investigated in powered studies. Such studies may also facilitate the evaluation of different cut-off values for FISH interpretation to improve the diagnostic accuracy for UTUC detection. In addition, a comparative assessment of multiple urine-based markers may be performed directly to investigate further optimization of the diagnostic pathway.

## Conclusion

UTUC detection is feasible with UroVysion^®^ FISH in one mL of passively collected upper tract urine. From a technical point of view, FISH could be used as an outpatient triage test to decide if follow-up ureteroscopy following kidney-sparing surgery of UTUC is necessary. The initial estimate of the diagnostic accuracy of FISH seems comparable to the current literature of FISH assessment in selective urine samples of greater volumes. Further evaluation of the diagnostic accuracy of FISH for the suggested diagnostic pathway should be stimulated.
